# Developmental aspects of the tympanic membrane: Shedding light on function and disease

**DOI:** 10.1002/dvg.23348

**Published:** 2019-11-25

**Authors:** Mona Mozaffari, Dan Jiang, Abigail S. Tucker

**Affiliations:** ^1^ Centre for Craniofacial and Regenerative Biology King's College London, Guy's Hospital London UK; ^2^ ENT Department Guy's Hospital London UK

**Keywords:** development, middle ear, tympanic membrane

## Abstract

The ear drum, or tympanic membrane (TM), is a key component in the intricate relay that transmits air‐borne sound to our fluid‐filled inner ear. Despite early belief that the mammalian ear drum evolved as a transformation of a reptilian drum, newer fossil data suggests a parallel and independent evolution of this structure in mammals. The term “drum” belies what is in fact a complex three‐dimensional structure formed from multiple embryonic cell lineages. Intriguingly, disease affects the ear drum differently in its different parts, with the superior and posterior parts being much more frequently affected. This suggests a key role for the developmental details of TM formation in its final form and function, both in homeostasis and regeneration. Here we review recent studies in rodent models and humans that are beginning to address large knowledge gaps in TM cell dynamics from a developmental biologist's point of view. We outline the biological and clinical uncertainties that remain, with a view to guiding the indispensable contribution that developmental biology will be able to make to better understanding the TM.

## 1 INTRODUCTION

**Figure 1:**
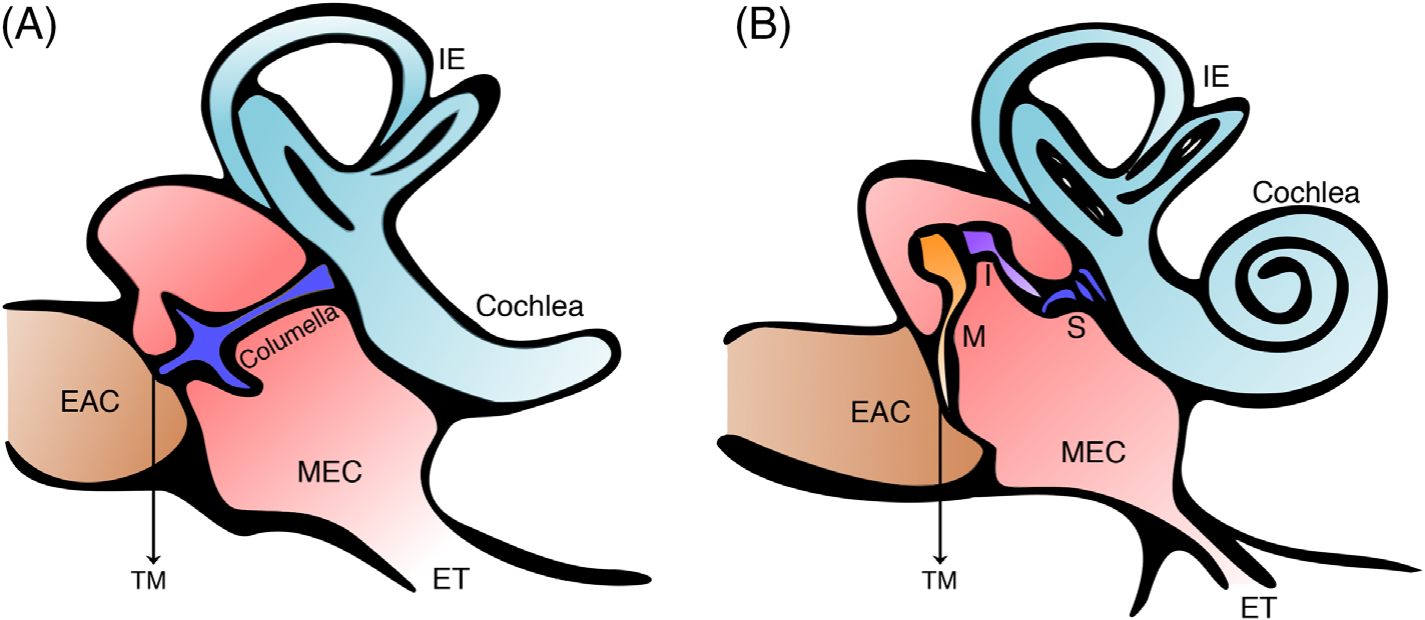
Middle ear anatomy. (a) Schematic of a sauropsid (e.g., bird or lizard) showing a single columella (or stapes) in the middle ear cavity. (b) Schematic of a mammalian middle ear showing a three‐ossicular chain connecting the tympanic membrane to the cochlea. EAC, external auditory canal; ET, eustachian tube; I, incus; IE, inner ear; M, malleus; MEC, middle ear cavity; S, stapes; TM, tympanic membrane

The role of the middle ear is in essence the same across all land vertebrates: an impedance mismatch corrector that transmits sounds from an air‐filled environment to a fluid‐filled cochlea (Figure 1). In therian mammals (placentals and marsupials) the pinna funnels vibrations into the ear canal (external auditory canal or EAC) where sound waves are captured by the ear drum (tympanic membrane or TM). The egg laying monotremes have a similar set up without the pinna, suggesting a later evolution of this structure during mammalian evolution. The lateral‐most bone of hearing, the malleus, inserts into the TM, levering to allow amplified vibrations to disrupt cochlear fluid and stimulate the hair cells of the inner ear. This is the beginning of the neural pathway to the auditory cortex, responsible for hearing. The TM is evolution's key but poorly understood solution to hearing with our dense bodies in an aerial environment.

**Figure 2:**
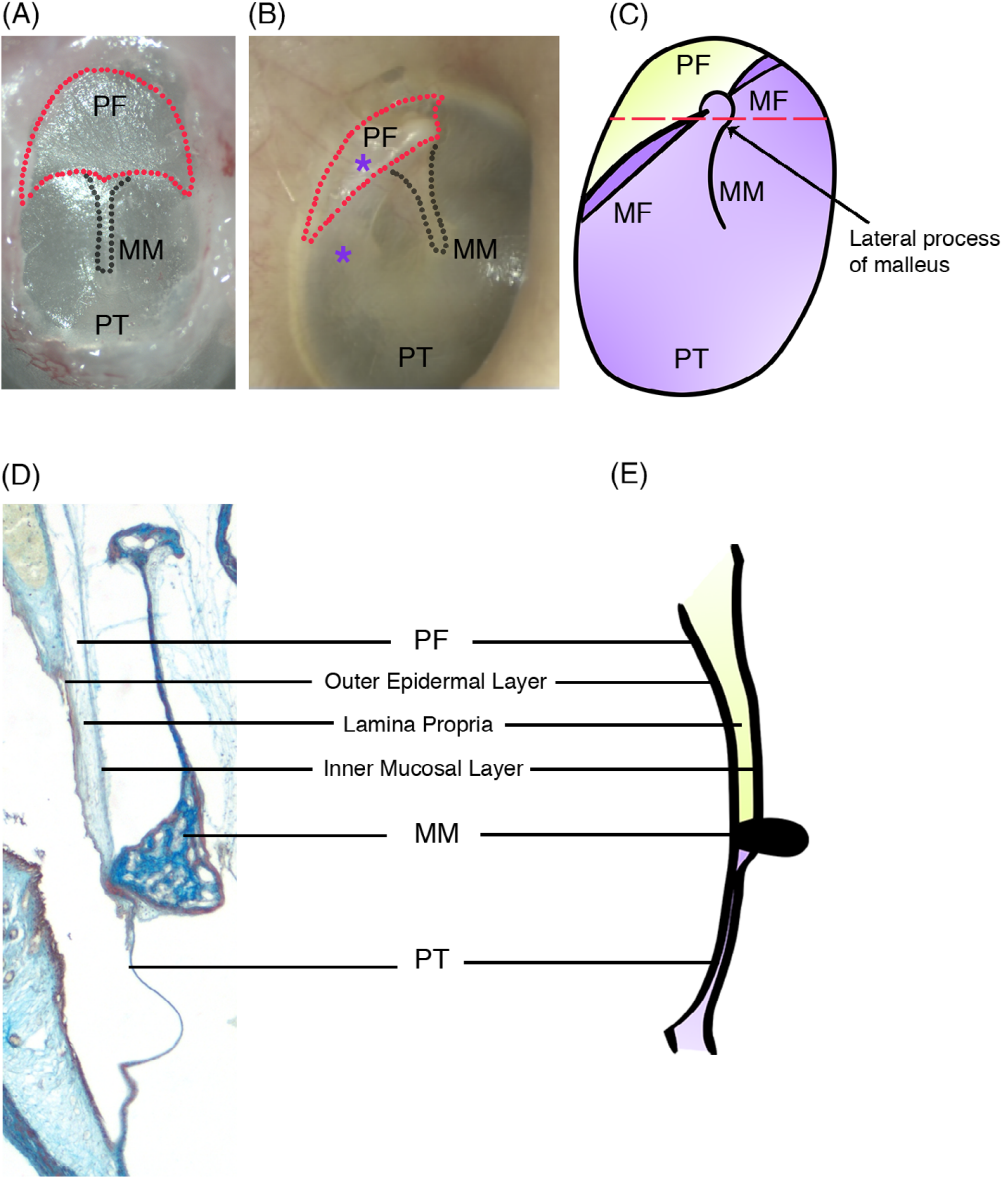
Tympanic membrane structure. (a) A murine tympanic membrane. The pars flaccida is broader and larger in comparison to (b) the triangular shaped pars flaccida of the human tympanic membrane. Asterisks denote areas of the eardrum most commonly affected by disease such as cholesteatoma or nonhealing perforations. (c) Schematic of human tympanic membrane. The pars flaccida and pars tensa are separated by the malleolar folds. The manubrium of the malleus inserts into and abuts the pars tensa. (d) and (e) Trichrome and schematic cross sections along dotted red line in (c) showing the three layers of the tympanic membrane with the pars flaccida being thicker than the pars tensa. MF, malleolar fold; MM, manubrium of malleus; PF, pars flaccida; PT, pars tensa

Unusually, disease affects the TM differently in its different parts, with the posterosuperior part being disproportionately affected by disease (R. K. Jackler, 1989; asterisks in Figure 2b). Intriguingly, this suggests that developmental aspects of the TM and the determination of its eventual anatomy, will be important in understanding acquired disease of the TM as well as congenital deformities.

The enigma typically driving developmental biology is how complex and diverse tissue emerges from a single primordial structure. This enigma is turned on its head when addressing TM development where uniquely all three germ layers, four if one considers the neural crest, converge to form a single membrane. Development of the TM is intricately and formatively related to that of the two structures it is flanked by, the ear canal and middle ear cavity. Interpreted through the prism of function and patterns of TM disease, recent advances in understanding middle ear development highlight important outstanding gaps in our knowledge of TM development, structure, and function.

### 1.1 TMs across land vertebrates

A tympanic (air filled) middle ear was initially proposed to be an ancient structure having evolved early on after vertebrates colonized land. In this scenario a mammalian middle ear would have evolved from a reptile middle ear. By the end of the 20th century a linear progression theory from reptilian to mammalian middle ear was largely refuted (Schnupp & Carr, 2009). High resolution micro‐computed tomography (μCT) capability allowed more detailed analysis of fossils, and it was postulated that a tympanic middle ear developed multiple times, independently and in parallel in different lineages (Clack, 1993; Schnupp & Carr, 2009). This means that the middle ear space we observe in birds and reptiles and some amphibians is not homologous to that of mammals, that is, is not a structure shared by the last common ancestor. The similarities we see in the TM across different classes of tetrapods therefore come from convergent evolution. The ossicles housed within the space also vary across land vertebrates. Unique to mammals is the three‐ossicular chain of the middle ear (malleus, incus, stapes) that act as a compound lever thought to allow for greater amplification of sound and the sensitive, high frequency hearing that distinguishes some mammals. In contrast, amphibians, reptiles, and birds possess a single ossicle, the columella, or stapes (Figure 1). The columella is homologous to the stapes of mammals, while the malleus and incus are homologous to the articular and quadrate, the key bones of the nonmammalian jaw joint and reflect the dramatic changes in articulation observed during mammalian evolution (Anthwal & Thompson, 2016; Tucker, 2017). Historically, mammals have been credited with better hearing, attributed to their three‐ossicular chain, with little evidence to back this up. The recent theory of independent middle ear evolution absolves the need to assign an advantage to having three ossicles over one. Interestingly, in experienced hands, total ossicular replacement prosthesis surgery where the entire ossicular chain is replaced with a single titanium rod, not unlike a reptilian columella, can achieve a near complete air‐bone gap closure such that there is no conductive hearing loss. Although modeling in human temporal bones does suggest subtle gains from an intact ossicular chain (Nakajima, Ravicz, Merchant, Peake, & Rosowski, 2005). A near complete air‐bone gap closure can also be achieved in cases of type three tympanoplasty where the malleus and incus are removed and the TM is laid onto the stapes head (Okada et al., 2014). One can therefore be almost as good as three.

The ear drum, like the middle ear cavity, is also not homologous between mammals and birds and reptiles (Takechi et al., 2016). For example, the mammalian ear drum is supported by a membranous bone, the tympanic ring (TR), while the nonmammalian ear drum is supported largely by an endochondral bone, the quadrate. Here we concentrate on the mammalian TM: its structure, function, and development.

### 1.2 Structure of the mammalian TM: A tale of two parts

Often simplified to the point of obscuring important features, the TM is in fact a complex structure. The mammalian TM is made up of two parts, a superior pars flaccida draped over the ossicles and an inferior pars tensa (Lim, 1995; Figure 2c). In contrast, nonmammalian ear drums are made up of a single taut membrane (Saunders et al., 2000). The mammalian pars tensa plays a key role in sound conduction, with the manubrium of the malleus inserting into it and transmitting captured sound waves along the ossicular chain. The pars flaccida is a more ambiguous structure with a suggested pressure‐equilibrating role (Robert, Funnell, & Laszlo, 1982). For example, its size, shape, and thickness vary considerably across species with sheep having large, elliptical pars flaccida, equal in size to the pars tensa, while in monkeys and humans the pars flaccida is triangular and significantly smaller than the pars tensa (Lim, 1968; Shrapnel, 1832). The cause of this variation is unclear and presents intriguing questions as to the function of the pars flaccida and its evolutionary history. In the mid‐20th century, leading evolutionary opinion held that the mammalian pars flaccida was homologous to the TM of nonmammals, while the pars tensa was suggested to be a novel structure created as extra bones were incorporated into the middle ear (Westoll, 1945). This was partially based on structural observations revealing that similar to reptilian eardrums, the mammalian pars flaccida is thick with elastic collagen (Robert et al., 1982). The two‐part ear drum of mammals was therefore a consequence of their evolutionary history. Given the well‐supported current view that mammalian and reptile ears drums are not homologous, we can reexamine the pars flaccida and pars tensa in a new light and pose new questions about their distinct function in mammals. In fact, faced with developmental constraints, the pars flaccida may have evolved as a consequence of middle ear anatomy rather than for a specific functional role in hearing. Such an influence from developmental constraints in evolution are described elsewhere, for example, in variations in mammalian cervical vertebrae and the need for initiation of eyes to serve as organizers even in eyeless blind cave fish (Arnold, Amson, & Fischer, 2017; Tian & Price, 2005).

The TM is made up of three layers (Figure 2d,e). An outer epidermal layer, a middle fibrous layer known as the lamina propria, and inner mucosal layer of epithelium (Lim, 1968). The middle lamina propria layer also houses the vessels and nerves that supply the TM. Recent single‐cell RNA sequencing data, looking at the cellular makeup of the layers of the TM, supports this established histological knowledge of TM structure, showing the predictable presence of mesenchymal, endothelial, smooth muscle, and Schwann cell clusters in the middle and inner layers of the TM, as well as clusters of keratinocytes at varying stages of differentiation in the outer epidermal layer (Frumm et al., 2019). This three‐ply structure is maintained in both the pars flaccida and pars tensa with important differences in the cellular makeup of the middle lamina propria layer. Unlike the pars tensa, this layer in the pars flaccida has no organized radial and circular fibers, consisting instead of loosely arranged elastic collagen (Lim, 1995). A common misconception is that the pars flaccida is thinner than the pars tensa. In fact, while being more elastic, it is actually thicker (Lim, 1995; Figure 2d). Interestingly, the pars tensa is also not uniform in structure with its posterior–superior quadrant resembling the pars flaccida's looser, disorganized lamina propria layer (Paço, Branco, Estibeiro, & Oliveira Carmo, 2009). These structural differences mirror differences in patterns of TM pathology, which will be discussed later.

The pars tensa sits within a c‐shaped TR, its concentric circular fibers getting ever closer in an outward pattern until they form a tough ligamentous annulus, at the TR. The pars flaccida sits above the mouth of the c‐shaped TR. It attaches superiorly to the downwards facing semicircumferential edge (or scutum) of the temporal bone. Its outermost epidermal layer is continuous with the ear canal and its inner mucosal layer is continuous with the epitympanic mucosa of the middle ear. In this way, the pars tensa is held taut within a hoop and the pars flaccida hangs much more loosely. An interesting series of experiments investigating the effect of tension on human skin keratinocytes showed an upregulation in keratinocyte migration, likely via the ERK 1/2 pathways, with increasing tension (Lü et al., 2013; Lü et al., 2016). This difference in tension may contribute to different properties of the different parts of the membrane.

The pars tensa thickens superiorly and the pars flaccida thickens inferiorly. Their meeting point forms a ligamentous band that holds the lateral process of the malleus in place. These are the anterior and posterior malleolar folds that mark the division between the pars tensa and pars flaccida (Figure 2c). Interestingly, this area has recently been proposed to house a key stem cell population of keratinocytes with long‐term renewal capability (Frumm et al., 2019).

### 1.3 Cellular dynamics within the tympanic membrane: Teasing apart the layers

The TM is essentially a complex and in parts compound, for example, at the insertion of the manubrium, structure. Thus, structural detail of fiber arrangements, cellular components, and keratinization play an important role in understanding TM function and pathology.

#### 1.3.1 Stem progenitor cells in the outer epidermal layer

**Figure 3:**
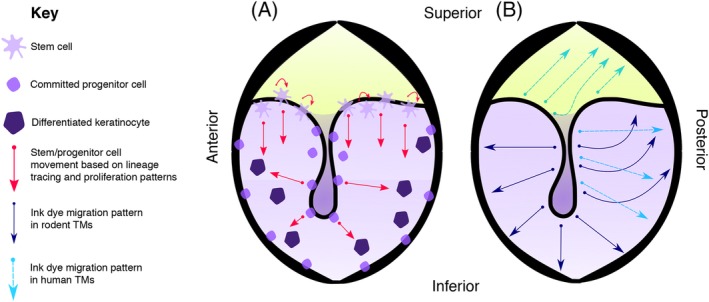
Cell dynamics in the outer epidermal layer of the tympanic membrane. (a) Location and movement of stem/progenitor cells based on lineage tracing experiments and proliferation assays (Frumm et al., 2019; Knutsson, von Unge, & Rask‐Andersen, 2011). (b) Migration patterns of keratinocytes based on ink dye labeling experiments in rodents and humans (Jackler, Santa Maria, Varsak, Nguyen, & Blevins, 2015; Michaels & Soucek, 1991). There is some variation in the reported pattern in humans (Alberti, 1964)

Early studies tracing dye markings show that the outer keratinizing layer of epidermis of the TM migrates in a specific pattern, radially from the manubrium in the pars tensa and posterior‐superiorly over the pars flaccida and the handle of malleus (L. Michaels & Soucek, 1991; Figure 3b). This lateral migration allows for the unique ability of the ear canal and ear drum skin to shed and migrate out of its anatomical cul‐de‐sac. This is quite unlike outer skin epidermis which stratifies outwards (Fuchs & Horsley, 2008). Due to this migratory potential of the outer keratin layer, and also because of easier experimental access to the outside of the eardrum, research has focused on progenitor/stem cell localization in the epidermal layer. Immunohistochemical analysis for integrins and cytokeratin 19 in rat and human TM indicated the localized presence of progenitor cells in the epidermal layer covering the manubrium, annulus, and handle of the malleus (Knutsson et al., 2011; W. Wang, Wang, & Tian, 2004). This finding was supported by studies investigating cell proliferation using the thymidine analogue BrdU. BrdU‐labeled cells were observed in the same areas as the integrins; along the handle of malleus and the annulus (Kakoi & Anniko, 1997). This finding has recently been confirmed using another thymidine analogue, EdU, in a single injection and chase experiment as well as a continuous label and chase experiment which additionally established a turnover time of 21 days for TM keratinocytes in mice (Frumm et al., 2019).

In an attempt to distinguish true stem cell niches from committed progenitor zones, Frumm et al., used live cell imaging of explanted Ki67 and Keratin 5 (K5) conditional cre mouse TMs to investigate in further detail the migration patterns and timing dynamics of TM epidermal keratinocytes in homeostasis. K5 is widely used as a marker for stem/progenitor cells in the epidermis. Intriguingly, these K5 positive keratinocytes appeared to follow a migratory superior–inferior path emanating from the malleolar folds, where the pars tensa and pars flaccida meet (Figure 3a). A similar migration pattern was observed in Ki67‐CreERT2; mTmG TMs. Using confetti reporter mice, with the above cre lines in vivo, clonal units of keratinocytes were evident in the TM epidermis, streaking downwards from the malleolar folds over a 3‐month period (Frumm et al., 2019). This is in contrast to the radial movement seen in historic dye studies (Alberti, 1964; R. K. Jackler et al., 2015; L. Michaels & Soucek, 1991; Figure 3b), which may have been confounded by inadvertent injury with the placement of dye or may have been reflecting migration from committed progenitor zones, such as the manubrium. The authors pose the hypothesis that the area where the pars tensa and pars flaccida meet is a true stem cell niche, that is, the cells that maintain the TM epidermis reside in this area. This is in contrast to the cells that reside in the central TM, whose faster proliferation and migration rate marks them out as committed progenitor rather than stem cells (Figure 3a).

#### 1.3.2 Repair of the ear drum

These stem and progenitor populations have been predicted to play a role in repair of the ear drum, in addition to homeostasis. In a series of experiments also using EdU, control and perforated TMs were compared by quantifying cell proliferation in the epithelial and mesenchymal layer of the TM using K5 and vimentin as markers, respectively (Chari, Frumm, Akil, & Tward, 2018). These authors confirmed the presence of stem/progenitor cells around the manubrium migrating radially toward the annulus in intact eardrums. In response to acute perforation, there was an increase in newly proliferated cells in both epithelial and mesenchymal TM layers at the site of perforation and around the manubrium. Interestingly however, there were newly proliferated keratinocytes throughout the epidermal membrane, even at sites away from the hole, suggesting a role for long distance cell signaling in response to TM perforation (Chari et al., 2018). This pattern of distant proliferation was not observed in the mesenchymal layer. Stem cells are typically associated with neurovascular supply (Jones & Fuller, 2009). It is this middle layer in which the nerves and blood vessel of the TM are encased and therefore a result suggesting less involvement in regeneration from this layer is surprising. Furthermore, it is commonly observed clinically that a perforated TM heals its three‐ply structure upon an initial epidermal scaffold (Johnson, Smallman, & Kent, 1990). While the lamina propria with its collagenous content appears a likely scaffolding layer, experiments in rodents suggest that repair starts with the epithelial layer, repairing inwards (Araújo, Murashima, Alves, Jamur, & Hyppolito, 2014; Yilmaz et al., 2019). It appears likely that the regenerative zones in the outer TM layers receive signals from the middle mesenchymal layer. A role for Pdgf signaling has been recently suggested in this context (Frumm et al., 2019). The role of the middle mesenchymal layer in TM regeneration remains an intriguing avenue for research.

#### 1.3.3 The unexplored inner mucosal layer

The regenerative role and progenitor/stem cell composition of the inner mucosal layer of the TM remains largely unexplored despite its potential significance in our understanding of middle ear disease. Lineage tracing experiments suggest that the inner layer of the pars tensa is derived from first pouch endoderm and is thus continuous with middle ear mucosa (Thompson & Tucker, 2013). Keratin 5 (K5) positive cells have been identified in the basal layer of the middle ear mucosa and play a role in adult middle ear epithelial maintenance (Luo et al., 2017; Tucker et al., 2018). Interestingly, a combination of scRNAseq and lineage tracing has highlighted Keratin 19 (K19) as a marker of mucosal stem/progenitor cells in the murine TM, rather than K5 (Frumm et al., 2019). The TM mucosa may therefore have a distinct identity from the middle ear mucosa. Interestingly, an analysis of label retaining cells in the middle ear cavity showed a high concentration of labeled cells on the mucosal side of the TM around the annulus and manubrium (Tucker et al., 2018). Matching stem/progenitor cell populations may therefore exist in both the mucosa and outer epithelial layers. Given our knowledge of middle ear disease, further probing the pattern and migration behavior of these cells would be of particular interest. Difficult access to the inner layer of the TM has limited experiments thus far but would be possible using explanted TMs in culture. Emerging single‐cell RNA sequencing data is providing useful validation of mucosal cell markers that can be harnessed for further experiments (Frumm et al., 2019). Are there distinct patterns of stem cell population in the inner layer of the pars flaccida versus the pars tensa? Do they mirror differing embryological origins and patterns of healing and disease?

### 1.4 TM: Function

**Figure 4:**
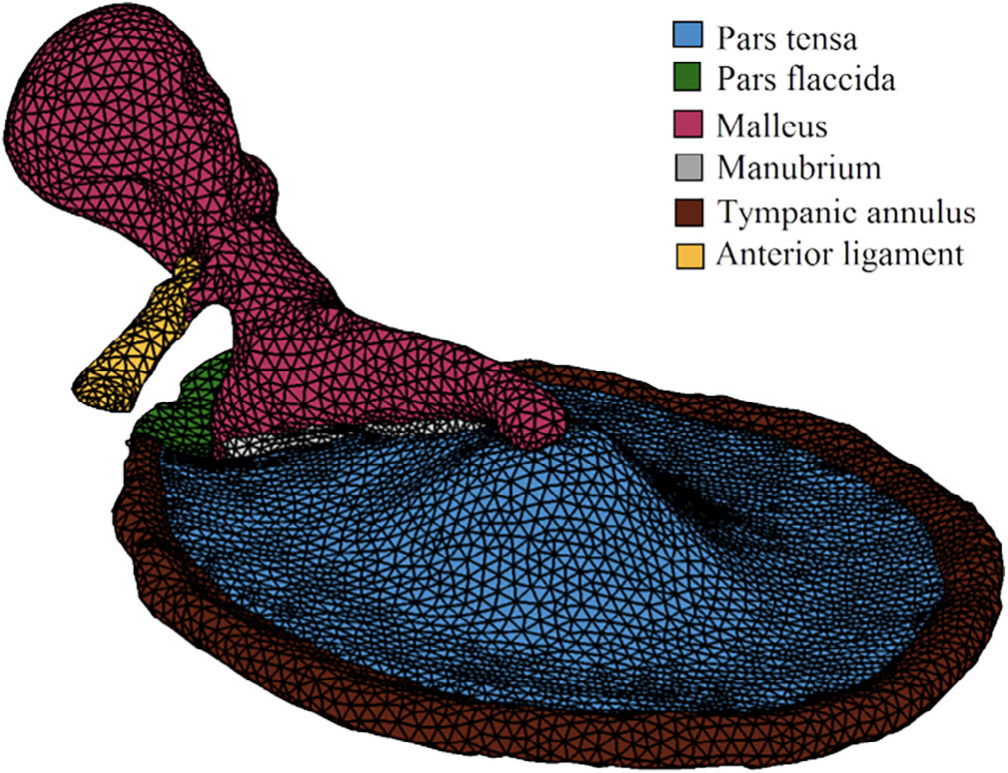
Example of a finite element model of a tympanic membrane (plus the malleus and its anterior ligament). A geometric mesh is used to model TM properties such as stiffness, allowing for predictive insights into how the TM may behave in response to sound waves. (Reprinted from Lobato, Paul, & Julio, 2018 with permission from AIP Publishing)

It is the very irregularities of the eardrum that make it an outstanding, and thus far unreplicated, loudspeaker. To describe it morphologically, the mammalian TM is far from a two‐dimensional circle. The pars tensa is shaped like a cone. The apex of the cone is formed by the insertion of the manubrium and points in toward the tympanum. Together with its radial collagen fibers, the lamina propria of the pars tensa is responsible for distorting sound in a bid to capture a broad range of frequencies (Gan, Feng, & Sun, 2004). These are collated at the manubrium and transmitted via the ossicular chain. The movement of the ear drum in response to sound is not a simple in and out but a complex wave, initiated in different parts of the membrane, which can be modeled with finite element analysis, defining the TM as a mathematical mesh based on its properties such as stiffness (Figure 4; Lobato et al., 2018; Volandri, Di Puccio, Forte, & Carmignani, 2011).

Given its high compliance, the role of the pars flaccida in sound conduction is not obvious. Immobilizing the ear drum of gerbils (who have a substantial pars flaccida) and measuring middle ear pressure in response to sound suggests that this part of the ear drum may play a role in pressure regulation (Robert et al., 1982). The middle ear cavity is connected to the nasopharynx via the Eustachian tube, which allows for gas exchange between the middle ear and airway. The Eustachian tube is opened and closed by muscles that line the tube, but in its resting state the Eustachian tube is closed, making the middle ear a confined air space. This causes problems with pressure regulation, a situation many of us have experienced when taking a plane journey. The flexibility of the pars flaccida has been suggested to allow it to move in and out of the middle ear space, thus mitigating pressure differentials, although there is no published evidence for this and indeed clinically when asking a patient to perform a Valsalva maneuver (directing pressured air into their middle ear), it is the pars tensa that bulges out. The pars flaccida could also play a role in protection against blasts that would cause rupture of a tight membrane. The size of the pars flaccida in different mammals might therefore relate to different needs for regulating pressure, or shock resistance. It is intriguing that sheep that have very large pars flaccida also have a behavior involving butting heads. In this case, the large pars flaccida may provide protection against perforation. An analysis of the embryonic origin and development of the pars flaccida versus the pars tensa may shed light on its hitherto ambiguous role.

As well as hearing, the eardrum plays an important barrier role, preventing items, and organisms from reaching the middle ear. In treating ear drum disease, the ear surgeon typically aims to achieve a “dry ear” over and above restoring its conductive properties (Warner, Burgess, Patel, Martinez‐Devesa, & Corbridge, 2009). While dry perforations are typically benign, those affected with chronic infection lead to a multitude of problematic and potentially devastating defects, discussed further below.

### 1.5 Development of the TM

**Figure 5:**
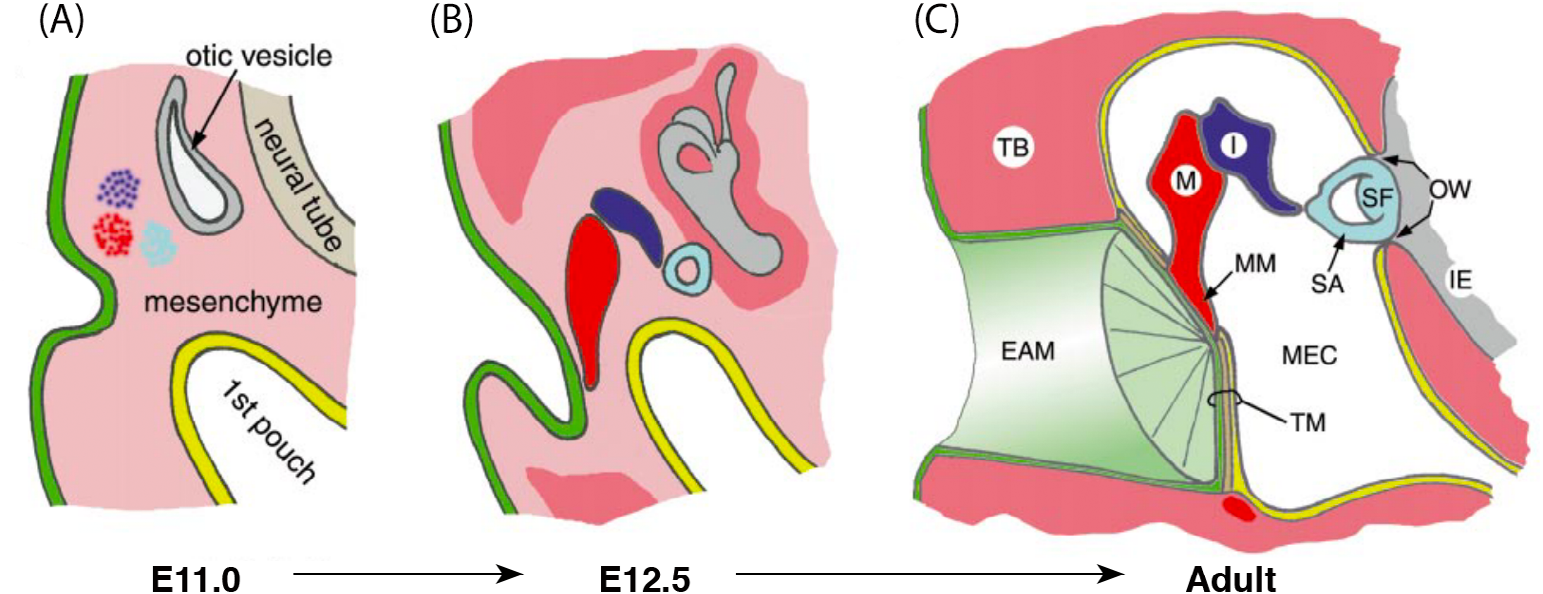
Development of the tympanic membrane. (a) and (b) Cells from the region of the first cleft invaginate toward the first pouch meanwhile the ossicles form within the neural crest cell derived mesenchyme. (c) The developing ectoderm‐derived ear canal and endoderm‐derived middle ear cavity sandwich a layer of mesenchyme between them forming a three‐layered tympanic membrane. (Modified and reprinted from Mallo, 2001 with permission from Elsevier). EAM, external auditory meatus; I, incus; IE, inner ear; M, malleus; MEC, middle ear cavity; MM, malleus manubrium; OW, oval window; SA, stapedius arm; SF, stapedius footplate; TB, temporal bone; TM, tympanic membrane

Until the turn of the century, understanding of ear drum development remained basic, relying on observational studies of the developing fetus and pathological specimens (M. S. Mallo, 2001). Experimental manipulation in birds shed some light on the mechanisms of development (Lomard & Hetherington, 1993) although as the structures are not homologous the relevance to mammalian development is unclear. This was built upon and brought to closer relevance to mammals with gene inactivation experiments in mice (Hofker & van Deursen, 2011). However, our understanding of ear drum formation still lacks detail. In basic terms, we still describe ear drum formation as a meeting of invaginating ectodermal cells from the region of the first pharyngeal cleft and the endodermal first pharyngeal pouch, sandwiching a layer of neural crest derived mesenchyme in between to form a three‐ply membrane (Figure 5). This description fails to address the TM in its two parts. How does the pouch navigate the ossicular chain to form the pars flaccida? If it does not, how does the pars flaccida form? It is likely, but not yet shown, that a de novo process of cavitation occurs superior to the ossicles which is separate to the cavitation process in the mesotympanum outlined by Thompson and Tucker (Thompson & Tucker, 2013). In part, in humans, even observational evidence of pars flaccida formation is lacking as attic cavitation has not occurred by 25 weeks and older gestational age samples are difficult to access (van Waegeningh, Ebbens, van Spronsen, & Oostra, 2019).

In recent years, we have gained more detailed understanding of the beginnings, the pharyngeal arch contributions, of this sandwiching process. Minoux et al., use Hoxa2 reporter and mutant mice to show that the EAC forms entirely within the Hoxa2‐negative first pharyngeal arch, not the cleft between the first and second arches as previously thought (Minoux et al., 2013). Furutera et al., further characterized TR formation in Hoxa2 knockout mice demonstrating that both the EAC and the TR, and so the whole TM, form within the first pharyngeal arch (Furutera et al., 2017). This is in contrast to the chick where in situ hybridization of Hoxa2 expression shows the TM to form entirely within the second pharyngeal arch; providing further evidence for independent evolution of a mammalian middle ear and ear drum. Interestingly, just as studies typically fail to address the ear drum as a structure formed of two distinct parts (a pars tensa and pars flaccida), the developing EAC is also typically referred to as a single structure, whereas we observe in the developing mouse and human two distinct parts, an outer cartilaginous part that is initially open and an inner bony part that forms as a closed plug (unpublished data). Studying the pharyngeal arch origin of the two separate parts may yield interesting results.

When something is more than the sum of its parts, it amounts to more than just the pieces that contributed to its formation. The phrase is apt for ear drum development. However, referring to the final structure of the ear drum and teasing apart its key components provides a good framework to discuss the developmental mechanisms involved in its formation, and emphasizes how precisely multiple structures need to interact to achieve the sum effect. To build an ear drum, an outer ectodermal and inner endodermal epithelial lining must meet and entrap a middle mesenchymal layer. The resulting sandwich must be taut and thin hanging within the c‐shaped TR. The superior part of this membrane, in the cradle of the c, must attach elsewhere. Importantly, the manubrium of the malleus must insert within the ear drum, to complete the piston effect so fundamental to air‐borne hearing. How much of this process do we understand?

The TR is thought to have a master role in ear drum formation. It forms early on in relation to outer and middle ear structures (E12.5 in mice) through a process of intramembranous ossification (Hsu, Chen, & You, 2017; Wilson & Tucker, 2004). Leading the EAC to it its correct position is thought to be under the control of the TR (Mallo & Gridley, 1996). Early experiments suggestive of this showed the EAC and TR to be mutually affected by teratogenic modification with retinoic acid (M. Mallo, 2000). In Gsc^−/−^ and Prx1^−/−^ mutant mice, which have been shown to lack TRs, an EAC is missing also (reviewed in Takechi et al., 2016). As well as genes encoding for morphogenetic processes, inactivating genes that encode signaling molecules such as Fgf8 and Endothlin1 disrupts both TR and EAC formation (M. Mallo, 2003). This suggests that signals from the first arch epithelium, including but perhaps not limited to, Fgf8 and Endothelin1 induce the expression of Gsc and Prx in some NCC‐derived mesenchymal cells causing them to form the TR. The relationship of the TR and EAM is further shown by loss of the transcription factor COUP‐TF11 in neural crest cells (Wnt1cre; COUP‐TFII^flox/flox^; Hsu et al., 2017). These mice have shortened and thickened TRs with the invaginating EAC heading in the right direction but going astray in the caudal extremity (Hsu et al., 2017). Thus, a complex system of interactions between the middle and outer ear guides the outer and inner linings of the ear drum to their destination at the TR.

While we know some mechanistic detail for the invaginating EAC, we know very little about the inner, endodermal side. How does the first pouch make its way toward the TR? It is likely that signals from the endoderm, such as SHH and BMP are involved, as shown in ossicular formation (Ankamreddy et al., 2019) but this has not yet been demonstrated.

An epithelial plug (the EAC) and a mesenchyme‐filled pouch meeting at the TR have yet to become a “membrane.” How does each side contribute a thin epithelial layer to the TM? Thompson and Tucker show that the endoderm pushes up against the TR and EAC. As the middle ear cavitates the endodermal wall is left behind forming the thin inner mucosal layer of the ear drum. The process of this retraction remains unclear. On the ectodermal, ear canal side, it is less clear still how a thin membrane remains. Opening on the ectodermal side does not appear to involve cell death but rather keratinization (Nishizaki et al., 1998). More interesting still, how is the middle mesenchymal layer taking shape in tandem with its flanking structures, and indeed the mechanical forces of the developing cranium?

To form a triad of interlinked and interdependent structures, the malleus manubrium (MM) joins the TR and external ear canal. It is imperative for correct function, that the MM inserts into the TM. A series of experiments by Mallo et al., suggest that the EAC plays an important role in this process. in vitro tissue recombination experiments indicate that the EAM is able to initiate chondrogenesis within mesenchymal tissue, indicating that in vivo the EAC alone could be inducing the manubrium and its placement within the TM (M. Mallo, 2000). Indeed, experimental conditions leading to nonformation of an EAC led to an underdeveloped manubrium despite a formation of a fully formed malleus. In contrast, a manubrium is seen to form in the presence of an EAC where the rest of the malleus is absent (M. Mallo, 2000). Similar findings have been found in patients, with defects in the ear canal correlating with defects in the manubrium, suggesting a conserved mechanism (Ishimoto, Ito, Kondo, Yamasoba, & Kaga, 2004).

### 1.6 TM: Pathology

The TM is affected by both acquired and congenital disease. The latter is less common and typically involves formation of a bony plate in place of a TM where there are coexisting external and middle ear deformities (Abdel‐Aziz, 2013). Given the propensity of the TM to be affected where there are canal and middle ear malformations, such congenital deformities of the TM are relatively common and highlight the intertwined development of these neighboring structures. Indeed, genetic mutations implicated in human middle ear congenital deformities, for example, Gsc and Eya1, have been shown to play inter‐related roles with other genes such as Prx1 and Fgf8 in middle ear development in mouse models (Parry et al., 2013; Rivera‐Pérez, Mallo, Gendron‐Maguire, Gridley, & Behringer, 1995; Tucker, Watson, Lettice, Yamada, & Hill, 2004).

Congenital deformity affecting the TM alone is very unusual. One example is congenital cholesteatoma of the ear drum. Cholesteatoma are expanding, keratin‐filled cysts which invade inwards to the middle ear with potentially devastating sequalae such as intracranial abscess. Mesotympanic congenital cholesteatoma, are found within the middle ear cavity and are thought to form from a remnant epidermoid cyst without involvement of the ear drum (L. Michaels, 1988). In contrast, TM cholesteatoma are confined to the ear drum, and at earlier stages only to the outer layer of the eardrum (Ching, Spinner, & Ng, 2017). Their etiology, and its relation to TM development, remains unknown. A third, much more common type of cholesteatoma, is acquired cholesteatoma and is discussed further below.

Acquired diseases of the eardrum are common, affecting patients of all ages. Unsurprisingly, perforations of this taut structure head the list. Perforations can be related to trauma, in which case they typically heal well, or they can be linked to infection, either acute suppurative otitis media or chronic suppurative otitis media. The latter heals notoriously poorly, even after surgical repair (Bhutta, Thornton, Kirkham, Kerschner, & Cheeseman, 2017). Until recently, an animal model for studying TM perforation was restricted largely to the Guinea pig, gerbil, and chinchilla, which provide accessible and sizeable TMs (A. Y. Wang et al., 2014). Experiments in mouse models offer the distinct advantage of using transgenic and reporter lines to better details cellular dynamics, as described above. The ability to maintain murine TMs in culture will provide even greater experimental choice (Frumm et al., 2019).

Relevant to this review, TM perforations behave differently based on their position, as well as size. Central perforations of the pars tensa heal well, whereas perforations of the pars flaccida or marginal perforations heal poorly (Warner et al., 2009). Dubbed “unsafe” by otologists, they demand regular inspection to check for the formation of acquired cholesteatoma. Interestingly acquired cholesteatoma do not form throughout the ear drum. These expanding and erosive cysts affect mostly the pars flaccida and posterior–superior quadrants of the pars tensa (R. K. Jackler, 1989). Their origin, aetioliogy, and indeed the mechanism behind their erosive qualities remain controversial. An explanation commonly accepted by otologists is that epidermal keratin once inside the middle ear cavity cannot be cleared and instead forms cysts which secrete erosive enzymes leading to further complications of hearing loss and intracranial infection (Persaud et al., 2018).

As well as a complication of TM perforation, acquired cholesteatoma can form as a consequence of a TM retraction pockets. Retraction pockets describe a condition where part of the TM lies deeper into the middle ear cavity than the rest of the TM. Their etiology remains contested. Most accepted is the negative pressure theory whereby poor eustachian tube function leads to in‐pulling of the TM. This is largely based on observational evidence such as the higher incidence of retraction pockets in patients with cleft palate (Parkes, Vilchez‐Madrigal, Cushing, Papsin, & James, 2018). Animal models where ligation of the eustachian tube leads to the formation of cholesteatoma support this theory also (Kim & Chole, 1998). A valid criticism of this theory is that cleft palate repair or the placement of a ventilation tube does not always prevent cholesteatoma. And indeed while eustachian tube dysfunction may be an initiating event, it does not explain the sustained growth of cholesteatoma. A newer theory poses middle ear mucosal traction and adhesions as a likelier culprit (R. K. Jackler et al., 2015). Further information on the structural origins and regenerative potential of the middle ear and TM will be invaluable in guiding this field of research. Like perforations, retraction pockets demand regular review to ensure they have not been complicated by cholesteatoma (Kakehata, Hozawa, Futai, & Shinkawa, 2005; Wells & Michaels, 1983). It is currently not possible to predict which retraction pockets are more likely to be affected. Given their high prevalence (one study found a quarter of a population of British school children to be affected) this poses a significant health economic burden (Maw, Hall, Pothier, Gregory, & Steer, 2011). Whether or not complicated by cholesteatoma, retraction pockets affect the superior and posterosuperior parts of the TM only (Sudhoff & Tos, 2000). A feature which may be explained by more detailed understanding of TM development and structure.

Less destructive than cholesteatoma but disabling nonetheless is granular myringitis of the ear drum. Resulting from trauma or chronic inflammation, it is characterized by a chronic painless otorrhea (ear discharge) and patches of deepithelialized TM. Again, the posterosuperior part of the ear drum is most affected. Little is known about the etiology of myringitis or indeed why only certain parts of the drum are affected.

“Etiology uncertain” too frequently follows discussions of TM pathology. Recent studies exploring the very basic cell physiology of the TM in homeostasis are beginning to shed light on the biological bases of TM disease. Having shed light on the variable migratory patterns of keratinocyte stem/progenitor cells in the epidermal TM, Frumm et al., pose the hypothesis that given minimal migratory activity of keratinocytes in the pars flaccida, keratinocytes within retractions here may be more likely to stratify and accumulate leading to cholesteatoma. Current understanding of acquired as well as congenital ear drum disease relies heavily on clinical observations and intuitive explanations. A better understanding of TM developmental biology will be invaluable in moving our knowledge forward.

## 2 CONCLUSION

The TM is fundamental to high quality, air‐borne hearing. Its formation captures much of the magic of developmental biology; how does a structure form amidst two cavities? And its developmental detail provides intriguing evolutionary clues. Clinically, its proximity to cranial structures equates to potentially devastating sequalae resulting from disease and thus better understanding of its development and function is imperative. With expanding genetic tools and cutting‐edge imaging technology so directly applicable and available to the developmental biologist, addressing the many unknowns surrounding ear drum form and function from a developmental biology point of view is more interesting and important than ever. Specifically, what do we need to know from development to help our understanding of TM disease? The TM is clearly made up of two distinct parts, the pars tensa and the lesser discussed and more pathology‐prone pars flaccida. How does the pars flaccida form? How does the ear canal open to expose the pars tensa and pars flaccida? What are its embryonic origins? How do retraction pockets start and why do they favor specific areas of the TM? How can we predict which will become complicated by cholesteatoma? Over two decades ago, gene inactivation experiments elucidated key aspects of ear drum development. A lull in research progress has ensued which appears on the cusp of reigniting.

## CONFLICT OF INTEREST

The authors have no conflicts of interest to declare.
